# The pathological features of hip abductor tendon tears – a cadaveric study

**DOI:** 10.1186/s12891-020-03784-3

**Published:** 2020-11-26

**Authors:** Mark F. Zhu, Brittany Smith, Sanjeev Krishna, David S. Musson, Peter R. Riordan, Sue R. McGlashan, Jillian Cornish, Jacob T. Munro

**Affiliations:** 1 Bone and Joint Laboratory, University of Auckland, 85 Park Road, Grafton, 1023 New Zealand; 2 Department of Orthopaedic Surgery, Auckland City Hospital, 2 Park Road, Grafton, New Zealand; 3 Department of Anatomy and Medical Imaging, University of Auckland, 85 Park Road, Grafton, New Zealand

**Keywords:** Hip abductor, Tendinopathy, Histology, Degeneration

## Abstract

**Background:**

The hip abductors are crucial in maintaining pelvic stability. Tears in these tendons are common and often debilitating. There is uncertainty regarding both the histological and macroscopic features of hip abductor tears. This study aims to clarify both the macroscopic and microscopic features of the tendon and enthesis in hip abductor tendon tears.

**Methods:**

Thirty-six cadavers with an average age of 81 were dissected, and the hip abductor mechanisms removed en-bloc. The presence, location and size of the tears were recorded and analysed. The samples were processed into histological blocks and viewed using both transmitted and polarised light. Tendon histology was graded using the modified Movin’s score in three sections (deep, middle and superficial layers) and the enthesis graded separately using 5-point criteria. Analysis of variance was used to confirm histological features associated with tears.

**Results:**

Tears were found in 24 of 36 samples (67%). The most common finding was an isolated tear in the gluteus minimus (46%), followed by concurrent gluteus medius and gluteus minimus tears (33%). Histology revealed significantly more degeneration in both the tendon (*p* = 0.0005) and enthesis (*p* = 0.0011) when tears were present. Furthermore, these changes were concentrated in the deeper layers of the tendon (*p* = 0.0002) and enthesis (*p* = 0.003).

**Conclusion:**

This study demonstrated degeneration as the primary pathology underlying hip abductor tendon tears. Degenerative changes occur in both the tendon and enthesis, with the deeper layers predominantly affected. These findings are important for guiding surgical repair techniques and to aid the development of novel materials and biologics.

**Supplementary Information:**

The online version contains supplementary material available at 10.1186/s12891-020-03784-3.

## Background

The gluteus medius and minimus are commonly referred to as the hip abductors. Tears of the hip abductor tendons are increasingly recognised as causes for chronic hip dysfunction and pain [1–4]. In the age group requiring hip arthroplasty, the incidence is approximately 20 to 25% [5–8].

Despite its prevalence, there is limited literature on the pathological features of disease [9, 10]. While the majority of surgical and imaging studies have described tears in the anterior gluteus medius, tears of the gluteus minimus and posterior gluteus medius have also been described [11]. Microscopically, degenerative tendinopathy is thought to be the underlying pathological change in hip abductor tendon tears. However, no previous studies were able to quantify and describe these changes in detail [1, 11].

In recent years, studies have attempted to improve the repair of both complete and partial tears of gluteus medius and minimus with limited success [12–14]. Conservative treatment with education and exercise programs is ineffective in patients with hip instability and full thickness tears [15, 16].

Further understanding of both the macroscopic and microscopic pathology are required to clarify the mechanism of failure and allow for more effective surgical repair. An understanding of the microscopic pathology will provide the basis for designing novel materials and biologics to improve repair outcomes. Therefore, the aim of this study is to use cadaveric tissues to determine the macro- and microscopic pathology associated with tears of the hip abductor tendons.

## Methods

### Specimens

Thirty-six hips in 30 cadavers (15 females and 15 males) were obtained from the Human Anatomy Lab, Department of Anatomy and Medical Imaging, University of Auckland. The cadavers were formalin-embalmed using Dodge anatomical mix (Dodge Chemical Corporation Incorporated). All tissue was donated with informed consent in compliance with the Human Tissue Act 2008 for teaching and research purposes. The average age of the cadavers was 80.9 years. No cadavers with a history of hip surgery on the dissected side were included in the study.

### Dissection, macroscopic examination and tissue processing

In all cadavers, the gluteus medius and minimus muscles and their attachment to the greater trochanter were removed en-bloc. Specimens were dissected to separate the gluteus medius from minimus. All superficial fat was removed, and the trochanteric bursa was carefully excised. The specimens were then examined macroscopically for tears in both the gluteus medius and minimus tendons. The presence, location (at the lateral facet or supero-posterior insertion for gluteus medius), size and type of tear (partial or complete) were recorded after careful dissection by a senior arthroplasty surgeon (JM) and the lead author (MZ). The width of the tear was measured parallel to the long axis of the footprint using a digital calliper. Other pathological features were noted; gross fatty atrophy of the muscle, bony changes at the footprint and tendon delamination.

**Fig. 1:**
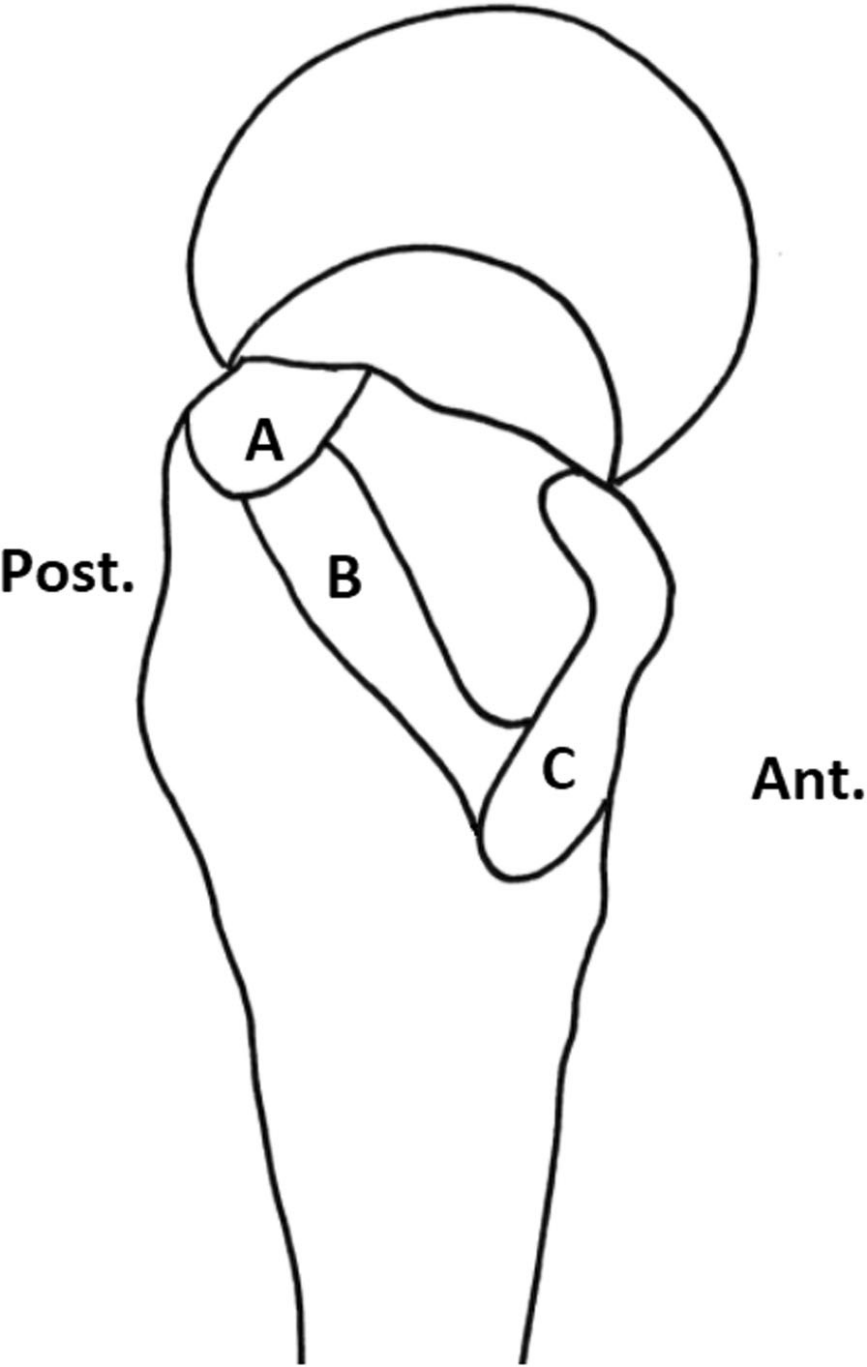
Illustration of the greater trochanter as viewed from the lateral aspect. (**a**) Supero-posterior facet/insertion; (**b**) lateral facet / insertion; (**c**) gluteus minimus insertion

Following macroscopic examination, the gluteus medius and minimus were dissected to capture the tendon, enthesis and its bony attachment (Fig. 1). For the gluteus medius, the sample was further split into the lateral and posterior insertions at the lateral and supero-posterior facets [17, 18]. These processed tissue blocks were then post fixed in 10% neutral buffered formalin for 5 days and decalcified in 10% formic acid for 14 days at 4 °C. Following decalcification, the tissue blocks were subjected to a routine dehydration process in 70% ethanol and embedded in paraffin wax. Sagittal sections of the distal 2 cm of tendon and the tendon bone junction were cut at 7 μm at 500 μm intervals throughout the tissue block. A total of 8 slides were obtained per sample and were stained using Haematoxylin and Eosin (H&E).

### Histological grading

**Fig. 2:**
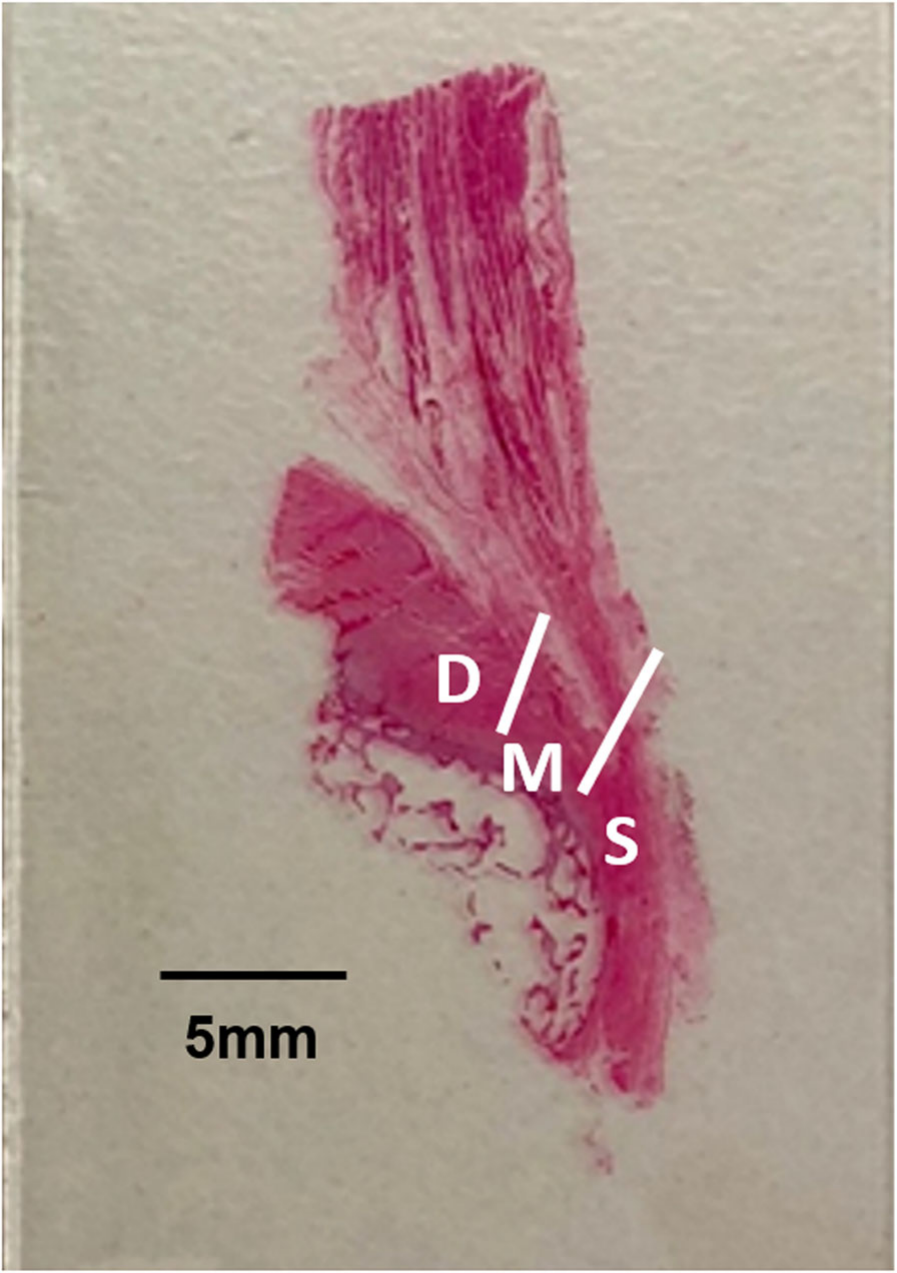
Cross section of anterior gluteus medius sample stained with H&E indicating the deep (**a**), middle (**b**) and superficial (**c**) areas for grading

Samples of the lateral and supero-posterior insertion of gluteus medius and the gluteus minimus were processed and graded separately. The slides of tissue were viewed under a microscope using transmitted and polarised light. The boundary between the tendon and enthesis was demarcated by the last longitudinal column of chondrocytic cells [19]. For the lateral gluteus medius insertion, the deep, middle and superficial layers of the tendon and enthesis were graded separately to identify differences in degeneration between the layers (Fig. 2). In all samples, the tendon was graded using the Modified Movin’s score (Additional file 1: Appendix 1) while the enthesis was graded using a 5 point scoring system derived from previous studies (Additional file 1: Appendix 2) [20–25]. In samples with complete tears, the region immediately adjacent to the tear was evaluated, as it would be otherwise impossible to grade both the tendon and enthesis. A minimum of 3 slides were viewed for each sample. For the lateral gluteus medius insertion, an overall score of the sample was obtained by averaging the score in the three layers. All samples were graded independently by two individuals with experience in bone and tendon research and reviewed by a musculoskeletal pathologist.

### Statistical analysis

All statistical tests were performed using GraphPad PRISM software (GraphPad Software, San Diego, CA). Comparisons of macroscopic features were performed using one-way analysis of variance where appropriate.

For microscopic features, degeneration scores were compared using Mann-Whitney’s U test between two groups and by one-way analysis of variance for three or more groups. Categorical variables were analysed using Fisher’s exact test. Interactions were assessed using two-way analysis of variance. Bonferroni correction was used when multiple comparisons were made. A *p* value < 0.05 was considered significant. The datasets used and analysed during the current study available from the corresponding author on reasonable request.

## Results

### Macroscopic features

Of the 36 samples, 24 (67%) had tears present in the hip abductors (either the gluteus medius, minimus or both). Of these 24 samples with tears present, 11 (46%) were isolated gluteus minimus tears, 5 (21%) were isolated gluteus medius tears and 8 (33%) involved the tendon of both muscles. Overall, the most common macroscopic finding was an isolated gluteus minimus tear, followed by tears involving both the gluteus minimus and medius.

**Fig. 3:**
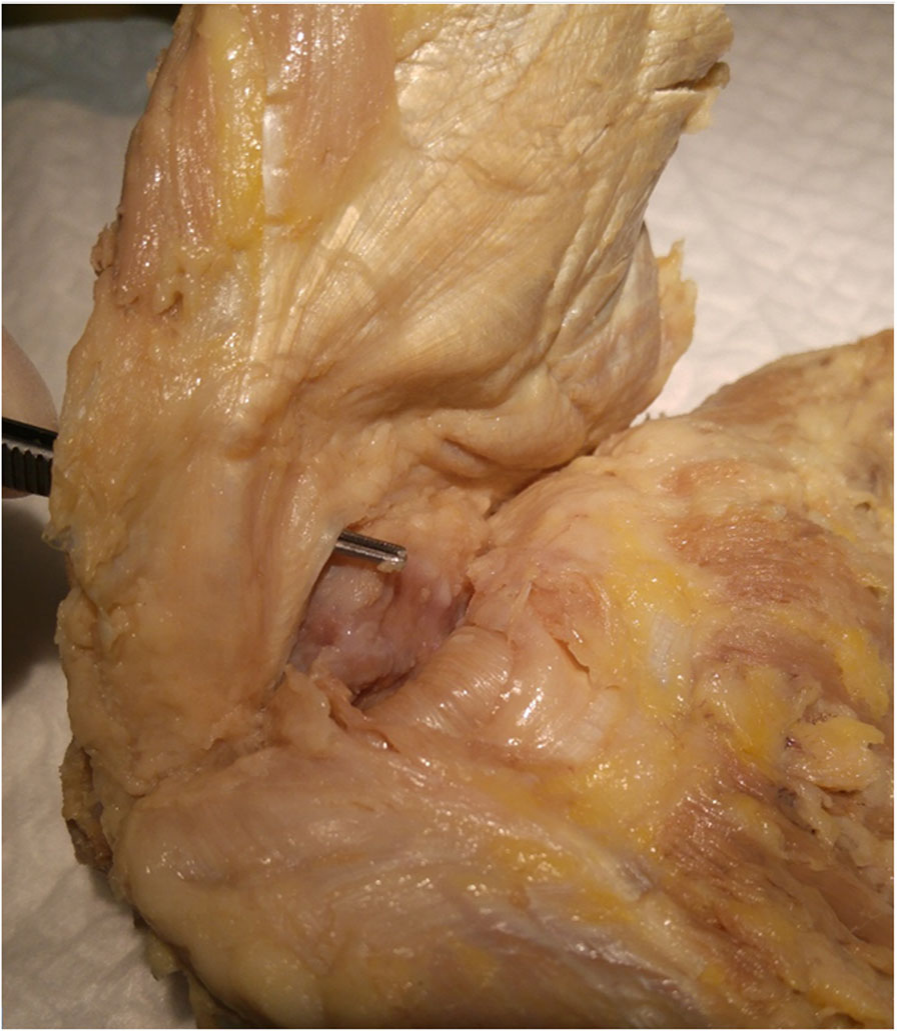
En-bloc sample showing complete tear through gluteus minimus and a partial tear through the overlying gluteus medius

In the 13 samples where a gluteus medius tear was identified, 11 tears were found in the anterior/middle portion of the tendon which inserts at the lateral facet of the greater trochanter. Only 2 small partial tears were found in the posterior portion of the gluteus medius. This portion inserts separately through a cord-like tendon at the supero-posterior facet of the greater trochanter. In both cases, the tears were less than 5 mm and no other abnormalities were identified in either the gluteus medius or minimus. In the gluteus minimus, all tears were found anteriorly, adjacent to the attachment of the gluteus medius tendon (Fig. 3).

Both complete and partial tears were found in the gluteus medius and minimus tendons. Partial tears were only observed on the under surface of the tendons. All tears were found in the distal 1 cm of tendon, while no tears were observed in either the gluteus medius or minimus tendons proximally. Interestingly, three samples with a complete gluteus minimus tear were also associated with partial under surface tears in the gluteus medius tendon immediately adjacent to the gluteus minimus tear.

The average width of a complete gluteus medius tear was 15.6 mm (SD 7.2) and 9.8 mm (SD 6.5) for partial tears. The average width of a complete gluteus minimus tear was 7.9 mm (SD 4.3) and 6.5 mm (SD 3.7) for partial tears. The differences in size between complete and partial tears was not statistically significant (*p* = 0.24).

Other macroscopic features were compared between samples with and without tears (Table 1). Of samples with complete tears, 89% were observed to have gross fatty atrophy of the related muscle. Delamination of the tendon was observed predominantly in samples with complete or partial tears. Bony changes (spur formation) were only observed in one sample with a complete tear.

**Table 1:** Associated features of hip abductor tendon tears

	Complete tear	Partial tear	Normal	*p*-value
Fatty atrophy of muscle	8 (89%)	2 (14%)	0	< 0.001
Delaminated	5 (56%)	8 (57%)	1 (10%)	0.030
Bony changes	1 (11%)	0	0	NS

*NS* not significant

### Microscopic features

#### Lateral insertion of gluteus medius

**Fig. 4:**
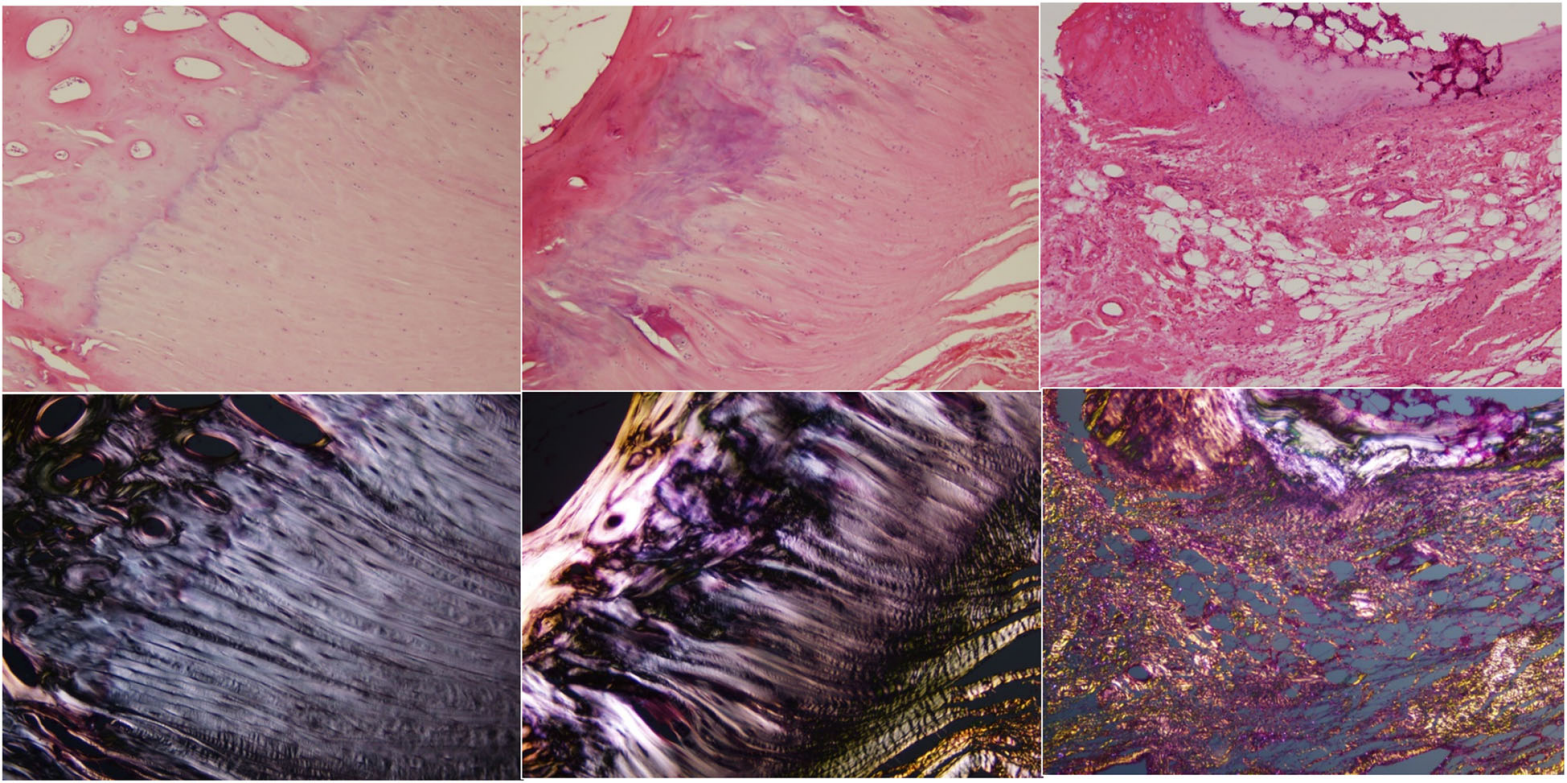
H&E stained slides through the gluteus medius bone tendon junction. Top images transmitted light, bottom images polarised light. Left - normal sample, middle - area adjacent to partial tear, right - area of complete tear. Samples demonstrate progressive degenerative changes

Comparisons were made between normal samples, samples with partial tears and samples with complete tears. The Modified Movin’s score was significantly different in normal (3.2), partial tear (8.6) and complete (8.9) tear samples (ANOVA *p* = 0.0005) (Fig. 4). Further post-hoc analysis separately demonstrated higher degeneration scores in the samples with complete (*p* = 0.0049) and partial tears (*p* = 0.0099) when compared to those with no tears (Table 2). The difference in degeneration between complete and partial tears was not statistically significant.

**Table 2:** Histology tendon and enthesis scores for lateral insertion of gluteus medius

	Tendon (SD)	p-value (post hoc)^a^	Enthesis (SD)	P-value (post hoc)^a^
Complete Tear	8.9 (2.0)	0.0049	7.1 (1.5)	0.011
Partial Tear	8.6 (1.7)	0.0099	7.4 (1.9)	0.0075
No Tear	3.2 (1.8)	reference	3.0 (2.0)	reference
p-value (ANOVA)	0.0005		0.0011	

^a^Post hoc for multiple comparisons

In the enthesis, the semi-quantitative degeneration scores (Additional file 1: Appendix 2) in normal (3.0), partial (7.4) and complete (7.1) tear samples were significantly different (ANOVA p = 0.0011). Again, post-hoc analysis demonstrated significantly higher scores in samples with both complete (*p* = 0.011) and partial tears (0.0075) when compared to those with no tears (Table 2).

When evaluating the degeneration scores in the superficial, middle and deep layers of the tendon, no significant difference between the layers were found in samples without tears (*p* = 0.19). However, in samples with complete and partial tears, the deep and middle layers scored significantly higher when compared to the superficial layer (*p* = 0.0002, Table 3). A similar pattern was also seen in the enthesis, where samples with tears were found to have significantly higher degeneration scores in the middle and deep layers (Table 4). In the middle and deep layers, significant differences in degeneration scores were found between samples with tears and no tears, where this was not evident in the superficial layer of the enthesis.

**Table 3:** Histology scores for lateral gluteus medius tendon layers

	Superficial	Middle	Deep	p-value*
Tears	6.0 (2.4)	8.7 (2.2)	11.5 (3.0)	0.0002
No tears	2.0 (1.7)	3.4 (1.6)	4.2 (2.5)	0.19
p-value^+^	0.0018	0.00050	0.00031	

^+^ *p*-value for tears vs no tears for each tendon layer

*p-value for difference in layers for samples with and without tears

Two way ANOVA - column factor (tendon layer) *p* = 0.0002, row factor (presence of tears) *p* < 0.0001, interaction *p* = 0.14

**Table 4:** Enthesis scores for lateral gluteus medius layers

	Superficial	Middle	Deep	p-value*
Tears	4.0 (2.7)	5.9 (2.3)	8.3 (3.0)	0.0030
No tears	2.4 (1.8)	2.8 (2.2)	3.7 (2.6)	0.54
p-value^+^	0.28	0.0029	0.0004	

^+^ *p*-value for tears vs no tears for each tendon layer

*p-value for difference in layers for samples with and without tears

ANOVA - column factor (tendon layer) *p* = 0.014, row factor (presence of tears) *p* < 0.0001, interaction *p* = 0.0815

Two-way ANOVA was unable to detect a significant interaction between layer examined and the presence of tears in either the tendon (interaction p = 0.14) or the enthesis (interaction *p* = 0.082).

#### Supero-posterior insertion of gluteus medius

In both samples where an isolated partial tear was found in the supero-posterior insertion, minimal degenerative changes were observed; in one sample, minimal regional variation in cellularity was found in the mid-substance of the tendon. The other sample displayed mild nuclei rounding and vascular infiltration, again in the mid-substance of the tendon. The enthesis of both samples exhibited no degenerative changes.

In samples of the supero-posterior insertion where a concurrent lateral insertion tear was present, the average degeneration score was 4.9 (SD 2.6) in the tendon and 3.8 (SD 2.2) in the enthesis. In contrast, the average degeneration scores in the same samples at the lateral insertion was 9.0 (SD 1.7) in the tendon and 7.6 (SD 2.0) in the enthesis. These differences were statistically significant (*p* = 0.012 and *p* = 0.013 respectively). No degenerative changes were found in the supero-posterior gluteus medius when tears were not present in the lateral portion of gluteus medius.

#### Gluteus minimus

In samples where a complete or partial tear were identified, the average modified Movin’s score was 13.4 and 11.1 respectively compared to 3.0 in normal samples. These differences were statistically significant (*p* = 0.0095) (Table 5). Again, enthesis involvement was seen in all samples with tears. The average enthesis degeneration score was 10.5 in complete tears, 9.6 in partial tears and 3.3 in normal samples (*p* = 0.021).

**Table 5:** Histology and enthesis scores for gluteus minimus insertion

	Tendon (SD)	p-value (post hoc)	Enthesis (SD)	p-value (post hoc)
Complete Tear	13.4 (1.7)	0.012	10.5 (1.7)	0.025
Partial Tear	11.1 (2.4)	0.2	9.6 (1.1)	0.16
No Tear	3.0 (1.8)	reference	3.3 (1.5)	reference
p-value (ANOVA)	0.0095		0.021	

## Discussion

This study is the largest series to date to examine hip abductor tendons in a cadaveric population. We demonstrated that the majority of tears involved the insertion of anterior/middle gluteus medius and the gluteus minimus. Microscopically, degeneration at both the tendon and enthesis underlies the observed macroscopic tears. Lastly, we found more advanced degeneration in the deeper layers of the tendon and enthesis, suggesting a potential pathomechanism of gluteus medius tears.

While many previous studies reported tears in the anterior gluteus medius, only a few have examined the gluteus minimus [26–28]. In the present study, gluteus minimus tears were observed more commonly than gluteus medius tears. Furthermore, all complete tears of the gluteus medius were associated with a concurrent gluteus minimus tear. This finding has clinical importance, as both ultrasound and MRI are poor at identifying gluteus minimus tears when a gluteus medius tear was also present [1, 26].

The pattern of disease identified also provides evidence that hip abductor tears may originate from the gluteus minimus. Previous electromyography and functional studies found increased loads in the gluteus minimus during the single stance phase of gait, particularly as the hip moves into adduction and extension [29, 30]. This “overload” phenomenon predominantly affects the gluteus minimus and not the gluteus medius [31]. Furthermore, the area of gluteus minimus deep to gluteus medius may experience increased compressive loads [8, 32]. This combination of increased tensile and compressive forces is a known risk factor for the development of tendinopathy and tears [32–34]. Functionally, the gluteus minimus acts synergistically with the anterior gluteus medius to stabilise the pelvis during gait and facilitate forward contralateral rotation of the pelvis [29, 30, 35, 36]. This shared function may also help to explain the large number of tears observed in both the gluteus minimus and the adjacent anterior gluteus medius tendons. In the current study, the majority of tears were found at the lateral insertion of the gluteus medius, with only two minor tears found at the supero-posterior insertion. This suggests that the supero-posterior insertion functions separately and is not involved in the degenerative process observed at the lateral insertion.

We identified a strong association between complete tendon tears and fatty atrophy of the gluteus medius and minimus. This has been reported in a number of imaging studies [37–39]. Once established, muscle atrophy is often irreversible and therefore, repair of the tendon may result in poor clinical improvement post-surgery and must be undertaken with caution [40, 41]. This raises the importance of early detection and repair of partial gluteus medius and minimus tears.

Microscopically, the present study has confirmed degeneration as the underlying pathology in hip abductor tendon tears. This is similar to histological findings in rotator cuff, Achilles and patella tendon tears [24, 42–44]. In the hip abductor tendons, a study by Fearon et al. examined tendon samples obtained intraoperatively at the time of gluteus medius repair. Given the small number of samples, they were only able to report qualitative degenerative change. Furthermore, the degree of degeneration was not specified and the enthesis was not examined [1]. Using semiquantitative scores, we found significant degeneration in not only the tendon but also the enthesis when tears were present in both the gluteus medius and minimus. Given the importance of the enthesis in load distribution, it is not surprising that degeneration in both tissue types contribute to tendon tears [21]. Interestingly, we were unable to demonstrate significant degenerative changes in two small, partial tears in the posterior insertion of gluteus medius. However, a larger sample size is required for definitive conclusions.

An important finding of the study was that microscopically, degenerative changes are concentrated in the deeper layers of the tendon and enthesis. In both tissue types, samples with tears exhibited higher degeneration scores in the deep and middle layers compared to the superficial layer. This difference was not seen in samples without tears. However, two-way ANOVA did not find a significant interaction between tear presence and tendon layer degeneration. This is likely due to early degenerative changes that maybe be present in macroscopically normal tendons. Degeneration in the deeper layers of the tendon (articular side) have also been observed in rotator cuff tears [44, 45]. The hip abductor and the rotator cuff share a number of similarities in their anatomy and function [5, 8, 36]. As such, the intrinsic mechanism of rotator cuff tendinopathy may apply to hip abductor tendon tears. The degeneration seen in the deeper layers may be secondary to the deeper fibres experiencing increased mechanical loads compared to the outer fibres, especially in adduction and small angles of abduction [32]. This theory is supported by both intraoperative findings in both the hip abductor and the rotator cuff [46–48]. Histologically, rotator cuff tears exhibit significantly more advanced degeneration in the deeper layers of the tendon [21, 44, 47, 48]. This again mirrors the findings of our study. Given the similarities between hip abductor and the rotator cuff tears, novel surgical methods, materials and biological augments maybe equally applicable to both problems.

With the supero-posterior insertion of gluteus medius and the gluteus minimus insertion, we did not grade layers of the samples separately. This was due to the smaller cross-sectional area of these insertions compared to the lateral insertion of gluteus medius. Future studies evaluating these areas may employ longitudinal sectioning to better appreciate the difference in degeneration between the layers [44].

A limitation of our study is that the tissue samples were obtained from cadavers with incomplete past medical histories. From patient discharge summaries and death certificates that were available, it was not possible to ascertain accurate information regarding the presence or absence of lateral hip pain and gait abnormalities. However, cadaveric studies have several advantages. Firstly, detailed macroscopic examination was possible as the area of interest was removed en-bloc. The insertion can be closely examined from both its superficial and deep surfaces without field of view limitations. Secondly, we were able to identify degenerative changes in both the tendon and enthesis by detailed histological examination of the entire tendon-bone unit. This provided greater insight into the pathological basis of disease which is not possible with imaging studies.

Our results have numerous implications for surgical repair. Firstly, we confirmed that the majority of gluteus medius tears involved the deep surface of the tendon. Therefore, this area must be specifically examined and addressed during repair. Secondly, we found higher than expected incidence of gluteus minimus tears that often co-exist with gluteus medius tears. Therefore, during surgical repair of the gluteus medius, the gluteus minimus should be carefully examined and repaired if torn. Lastly, the development of new biological materials for tendon repair requires improved understanding of the pathological features of abductor tendon tears. In our study, we have visualised and identified both tendon and enthesis degeneration as the key feature in hip abductor tendinopathy. Hence, novel materials and biologics must target both tissue types to improve healing following surgery.

In conclusion, we found degenerative changes are the underlying pathology in hip abductor tendon tears. Both the macroscopic and microscopic features of degeneration were predominantly observed in the deep layers of the tendons. These findings are important for guiding surgical repair techniques and to aid the development of novel materials and biologics in order to improve surgical outcomes.

## Supplementary Information

**Additional file 1:** **Appendix 1.** Modified Movin’s score for tendinopathy. **Appendix 2.** Enthesis scoring criteria, maximum score 15, minimum score 0.

## Data Availability

All data generated during this study are included in this published article.

## Abbreviations

H&E: Haematoxylin and Eosin
ANOVA: Analysis of Variance
SD: Standard deviation
MRI: Magnetic resonance imaging
