# Supplementary material for: The pathological features of hip abductor tendon tears – a cadaveric study
Source: BMC Musculoskelet Disord. 2020 Nov 26;21:778. doi: 10.1186/s12891-020-03784-3 (PMC7690166; doi:10.1186/s12891-020-03784-3)
Supplement: Supplementary file 1 — Additional file 1: Appendix 1. Modified Movin’s score for tendinopathy. Appendix 2. Enthesis scoring criteria, maximum score 15, minimum score 0. [file 12891_2020_3784_MOESM1_ESM.docx]

**Appendix 1: Modified Movin’s score for tendinopathy**

| Items | Score |
| --- | --- |
| Fibre structure | Normal (0)  Slightly abnormal (1)  Abnormal (2)  Markedly abnormal (3) |
| Fibre distribution | Normal (0)  Slightly abnormal (1)  Abnormal (2)  Markedly abnormal (3) |
| Rounding of nuclei | Normal (0)  Slightly abnormal (1)  Abnormal (2)  Markedly abnormal (3) |
| Regional variations in cellularity | Normal (0)  Slightly abnormal (1)  Abnormal (2)  Markedly abnormal (3) |
| Vascularity | Normal (0)  Slightly abnormal (1)  Abnormal (2)  Markedly abnormal (3) |
| Decreased collagen stainability | Normal (0)  Slightly abnormal (1)  Abnormal (2)  Markedly abnormal (3) |
| Hylinization | Normal (0)  Slightly abnormal (1)  Abnormal (2)  Markedly abnormal (3) |

Maximum = 21, minimum =0

**Appendix 2: Enthesis scoring criteria, maximum score 15, minimum score 0**

| \| Items \| Score \| \| --- \| --- \| \| Fibre alignment (< 15 degrees from tendon alignment)^19^ \| Normal (0)  <25% abnormal (1)  25-50% abnormal (2)  >50% abnormal (3) \| \| Presence/number of tears^23^ \| Normal (0)  Minimal (1)  Moderate (2)  Marked (3) \| \| Tidemark score^3^ \| Normal (0)  Present but abnormal (1)  Markedly abnormal (2)  Absent (3) \| \| Regional variations in cellularity^4,5^ \| Normal (0)  Slightly Abnormal (1)  Abnormal (2)  Markedly abnormal (3) \| \| Regional variations in stainability^5^ \| Normal (0)  Slightly Abnormal (1)  Abnormal (2)  Markedly abnormal (3) \| |  |
| --- | --- | --- | --- | --- | --- | --- | --- | --- | --- | --- | --- | --- | --- |
